# Barriers to breast cancer treatment in Brazil: A study on migration and regional disparities^☆^

**DOI:** 10.1016/j.puhip.2025.100614

**Published:** 2025-05-13

**Authors:** Marcelo Antonini, Gabriela Moreira Santos, André Mattar, Marina Diogenes Teixeira, Andressa Gonçalves Amorim, Marina Fleury de Figueiredo, Marcellus do Nascimento Moreira Ramos, Francisco Pimentel Cavalcante, Eduardo Camargo Millen, Antonio Luis Frasson, Felipe Zerwes, Odair Ferraro, Fabricio Palermo Brenelli, Juliana Francisco, Luiz Henrique Gebrim

**Affiliations:** a Mastology Departament of Hospital do Servidor Público Estadual (HSPE-FMO), São Paulo, Brazil; b Mastology Departament of Women's Health Hospital, São Paulo, Brazil; c Mastology Departament of Hospital Geral de Fortaleza (HGF), Fortaleza, Brazil; d Breast Surgeon at Americas Oncologia, Rio de Janeiro, Brazil; e Breast Surgeon at Hospital Israelita Albert Einstein, São Paulo, Brazil; f Mastology Departament of Medical School of PUC-RS, Porto Alegre, Brazil; g Mastology Departament Universidade Estadual de Campinas, Campinas, Brazil; h Breast Surgeon at Prevent Senior, Sao Paulo, Brazil; i Mastology Departament Beneficência Portuguesa de São Paulo, São Paulo, Brazil; j BBREAST - Brazilian Breast Association Team, São Paulo, Brazil

**Keywords:** Breast cancer, Patient migration, Health disparities, Radiotherapy access, Cancer staging, Healthcare system

## Abstract

**Background:**

Breast cancer (BC) is one of the most prevalent cancers globally, with 2.3 million new cases annually in 2023. In Brazil, it is estimated that there are 74,000 new cases per year, representing 20.3 % of cancers in women in the country. Limited access to adequate treatment forces patients to seek care elsewhere, straining the healthcare system and delaying treatment initiation. This study aimed to determine the migration rate of BC patients in Brazil and specifically to analyze migration rate by stage and treatment modality, as well as its costs and possible reasons for delays.

**Study design:**

A cross-sectional observational ecological study was conducted using retrospective data on the prevalence of breast cancer in Brazil, including the number of diagnoses and treatments in each state. The study evaluated patient migration rate for treatment, with information obtained from the DATASUS - SISCAN/Cancer Information System database. The selected analysis period was from 2017 to 2022, during which all necessary variables were available.

**Results:**

Among the 275,140 cases analyzed, 98.18 % were women. The national migration rate was 2.12 %, ranging from 0.35 % in the Northeast to 9.31 % in the Midwest. Stage IV had the highest migration rate (1.74 %), and migration rate for radiotherapy was significant in some states, reaching 100 % in specific cases.

**Conclusion:**

The migration rate of BC patients for treatment in Brazil shows significant regional variations, with higher rates in the Midwest and lower rates in the South. Some states exhibited a 100 % migration rate for access to radiotherapy. Stage IV patients are the most prone to migrate, and the search for radiotherapy can lead patients to move to other states. These results highlight the need for specific health policies to ensure access to treatment across the country.

**Study adds**

• **Identifies regional disparities in breast cancer treatment**: This study provides detailed insights into the significant regional variations in the migration rate of breast cancer patients for treatment across Brazil, highlighting gaps in access to care.
• **Emphasizes the impact of cancer staging on patient migration rate**: The research reveals that patients with Stage IV breast cancer are more likely to migrate for treatment, particularly for access to radiotherapy, indicating a critical need for better resource distribution.
• **Quantifies the burden of radiotherapy access issues**: The study quantifies migration rates for radiotherapy, including cases where migration rates reaches 100 %, underlining the urgent need for policies to improve local access to essential cancer treatments.

**Implications for policy and practice**

• **Regional investment in oncology infrastructure**: The study underscores the need for targeted investments in regions with higher migration rates, particularly in the Midwest, to improve access to local breast cancer treatment, reducing patient travel and associated delays.
• **Expansion of radiotherapy services**: Given the high migration rates for radiotherapy, especially in Stage IV patients, expanding radiotherapy facilities in underserved areas could reduce patient burden and improve timely access to care.
• **Development of national strategies for equitable cancer care**: The findings support the creation of national health policies aimed at ensuring equitable access to cancer treatments, minimizing regional disparities and improving overall treatment outcomes across the country.

## 1 Introduction

In 2024, the Global Cancer Observatory (GLOBOCAN) report on the global cancer burden revealed that breast cancer was the most commonly diagnosed cancer in over 80 % of the world's countries (154 out of 185) and the leading cause of cancer-related deaths [1]. According to the latest GLOBOCAN estimates from 2023, breast cancer remains the most prevalent cancer worldwide, with 2.3 million new cases each year. In Brazil, the projected cancer incidence for the 2023–2025 period is 704,000 new cases, with 483,000 excluding non-melanoma skin cancers, and 74,000 cases of breast cancer. This represents 10.5 % of all cancer diagnoses and 20.3 % of cancers among women [2].

Following this concerning global trend, Brazil shows a continuous increase in breast cancer incidence rates. Excluding non-melanoma skin tumors, breast cancer is the most prevalent cancer among women in all regions of the country. For each year of the triennium 2023–2025, an estimated 73,610 new cases represent an incidence rate of 41.89 cases per 100,000 women [3], compared to an estimated 66,280 new cases in 2022 [4].

The age-adjusted breast cancer mortality rate in Brazil, based on the world population, was 11.71 deaths per 100,000 women in 2021. The Southeast and South regions have the highest rates (12.43 and 12.69, respectively), while the North has the lowest (8.59) [3]. A decline in mortality was observed in 2020 and 2021, possibly related to the pandemic, where COVID-19 deaths may have been a competing cause. Broadly, breast cancer deaths rank first in the country, representing 16.1 % of total cancer deaths. This pattern is repeated in all Brazilian regions except the North, where breast cancer deaths rank second at 13.7 % of women, only surpassed by cervical cancer [3,4]. This global and national trend underscores the urgency of continuous efforts to promote awareness, early detection, and access to therapeutic treatments, thereby reducing the disease's impact.

In response to the global burden of breast cancer, the World Health Organization launched the Global Breast Cancer Initiative (GBCI) in 2021, offering a structured framework to address breast cancer challenges in low- and middle-income countries such as Brazil [5,6]. The GBCI focuses on three critical pillars: early detection, prompt diagnosis, and treatment to completion. Each pillar has specific key performance indicators (KPIs) designed to measure progress and improve outcomes globally [[7], [8], [9]]. This initiative aims to reduce global breast cancer mortality by 2.5 % per year, thereby averting 2.5 million deaths from breast cancer globally between 2020 and 2040 [7].

The Unified Health System (SUS) is one of the largest and most complex public health systems in the world, providing services ranging from primary to tertiary care. Established by the 1988 Federal Constitution (CF-88), SUS guarantees healthcare as a fundamental right for all Brazilians, providing universal and free access. However, given Brazil's vast geographic size, living in remote areas far from major cities can pose significant challenges, particularly in terms of distance and limited access to public healthcare services, especially for tertiary care [10,11].

These challenges persist despite established principles of healthcare hierarchy and regionalization. Even among individuals with similar socioeconomic backgrounds, access to healthcare services is more limited in rural areas compared to urban centers. The greater difficulty in accessing services in rural regions is primarily due to the reduced availability of healthcare facilities, often requiring patients to travel longer distances for care [12].

The lack of access to adequate treatment, whether due to logistical or financial issues, contributes to unfavorable outcomes and poorer prognoses. Middle-income countries suffer a disproportionately high breast cancer mortality rate compared to its incidence, often due to late diagnosis and treatment [13].

This study aimed to determine the migration rate of breast cancer patients in Brazil and specifically to analyze migration rate by stage and treatment modality, as well as its costs and possible reasons for delays.

## 2 Methods

### 2.1 Study design

A cross-sectional observational ecological study was conducted using retrospective data on the prevalence of breast cancer in Brazil, including the number of diagnoses and treatments in each state. The study evaluated patient migration rates for treatment, with information obtained from the DATASUS - SISCAN/Cancer Information System database. The selected analysis period was from 2017 to 2022, during which all necessary variables were available. Our analytical framework is aligned with the pillars of the WHO's Global Breast Cancer Initiative (GBCI) [6], with a particular focus on treatment completion (Pillar 3), through the examination of regional disparities in access to different treatment modalities. In addition, we addressed the GBCI key performance indicator (KPI) for early detection (Pillar 1) by analyzing the distribution of clinical stages at diagnosis across Brazilian states [9].

### 2.2 Database

The study utilized data from DATASUS (Department of Informatics of the Unified Health System), an information system from Brazil's public health system (SUS). DATASUS is a governmental body responsible for collecting, processing, analyzing, and disseminating public health data in the country. The department maintains computerized systems that allow online research of all collected data. Within DATASUS, there are two complementary databases: SISCAN (Cancer Information System) and the Oncology Panel Brazil. SISCAN is used for the control and monitoring of cervical and breast cancer, while the Oncology Panel Brazil is an online platform that provides and consolidates all data on the cancer situation in Brazil. This information is public and can be accessed freely via the website https://datasus.saude.gov.br/informacoes-de-saude-tabnet/. It is important to note that the DATASUS database is frequently updated; therefore, the numbers presented and analyzed in this study refer to the data accessed on April 5, 2023.

### 2.3 Data validation and quality assessment

To ensure data quality, we implemented a systematic validation process for all extracted data. First, we cross-verified the consistency of records between the SISCAN and Oncology Panel Brazil databases, addressing any discrepancies by consulting additional DATASUS records. We also conducted logical validation checks, including identifying and flagging implausible values such as negative time intervals or impossible staging progressions.

The DATASUS database, while comprehensive, has known limitations regarding data completeness. To assess potential underreporting biases, we compared our dataset with cancer registry data from population-based cancer registries in selected cities. Our validation indicated approximately 85–90 % coverage of breast cancer cases treated in the public health system, with potentially higher underreporting in remote areas and lower-resourced regions, which may affect our estimates of migration rates, particularly for these areas.

### 2.4 Handling of missing data

Missing data presented a significant challenge, particularly for clinical staging information (missing in 14.2 % of records) and treatment modality details (missing in 9.7 % of records). We applied the following strategies to address these gaps: For records missing the state of diagnosis but having treatment location data, we used the treatment location as a proxy for the diagnosis location if no migration was indicated in other fields. Records with missing clinical staging were excluded from stage-specific analyses but retained for overall migration rate calculations. For treatment modality analyses, we analyzed only complete records rather than using imputation methods, as assumptions about missing treatment data could potentially introduce bias into our assessment of treatment accessibility.

### 2.5 Included patients and analyzed variables

We included data extracted from SISCAN between 2017 and 2022, using the ICD-10 code C50.9 (breast cancer) as a filter criterion. The sub-variables analyzed included the state of diagnosis, state of treatment, clinical staging, and treatment modality (chemotherapy, radiotherapy, and surgery). This evaluation resulted in a total of 275,140 patients.

In this study, 'migration rate' refers to the percentage of patients who received treatment in a different state from where they were diagnosed, quantifying inter-state movement for breast cancer care.

### 2.6 Statistical analysis

To evaluate statistical significance, Chi-Square tests were used to compare migration rate by clinical staging and migration rate by state, and Two-Proportion Z-tests were used to obtain p-values when comparing migration arte by treatment modality. All analyses were conducted using statistical software.

### 2.7 Ethics committee

As this is an ecological study, it is exempt from requiring.

## 3 Results

The results are presented in alignment with the WHO's GBCI framework [[4], [5], [6], [7]], focusing on regional disparities in early detection (Pillar 1) and treatment completion (Pillar 3), as well as barriers to accessing care across Brazil's diverse geographical landscape.

### 3.1 Regional distribution of breast cancer cases

The analysis of breast cancer case distribution across Brazil from 2017 to 2022 revealed marked regional disparities (Table 1). The Southeast region bore the highest burden, with São Paulo alone accounting for 61,682 cases (22.4 % of all national cases), followed by Minas Gerais with 32,437 (11.8 %) and Rio de Janeiro with 23,259 cases (8.5 %). The South region reported the second highest concentration, led by Rio Grande do Sul (23,658; 8.6 %), Paraná (19,593; 7.1 %), and Santa Catarina (12,336; 4.4 %). In the Northeast, Bahia recorded the most cases (15,219; 5.5 %), followed by Pernambuco (12,672; 4.6 %) and Ceará (11,331; 4.1 %). The Midwest presented moderate case volumes, with Goiás registering 6769 cases (2.4 %), while both Mato Grosso and the Federal District contributed approximately 1.1 % each. The Northern region consistently reported the lowest case counts, led by Pará with 4244 cases (1.5 %), followed by Amazonas (2406; 0.9 %) and Rondônia (1902; 0.7 %). Altogether, 275,140 breast cancer cases were documented nationally during the study period, underscoring substantial geographic disparities in disease burden across the country.

**Table 1:** Distribution of breast cancer cases in Brazil 2017 to 2022 by region and state.

Federative Unit	n	%
**Northern Region**		
Rondônia	1902	0.7
Acre	512	0.2
Amazonas	2406	0.9
Roraima	403	0.1
Pará	4244	1.5
Amapá	328	0.1
Tocantins	1051	0.4
**Midwest Region**		
Mato Grosso do Sul	3411	1.2
Mato Grosso	3102	1.1
Goiás	6769	2.4
Distrito Federal	3043	1.1
**Southeast Region**		
Minas Gerais	32,437	11.8
Espírito Santo	6258	2.3
Rio de Janeiro	23,259	8.5
São Paulo	61,682	22.4
**South Region**		
Paraná	19,593	7.1
Santa Catarina	12,336	4.4
Rio Grande do Sul	23,658	8.6
**Northeast Region**		
Maranhão	7836	2.8
Piauí	3129	1.1
Ceará	11,331	4.1
Rio Grande do Norte	6995	2.5
Paraíba	5160	1.9
Pernambuco	12,672	4.6
Alagoas	3270	1.2
Sergipe	2134	0.8
Bahia	15,219	5.5

Brazil	275,140	100

Legend: This table presents the distribution of breast cancer cases in Brazil, broken down by region and state, from 2017 to 2022. The data includes the number of cases (n) and the corresponding percentage (%) of the total cases.

### 3.2 Migration rate by state and region for breast cancer treatment

**Fig. 1:**
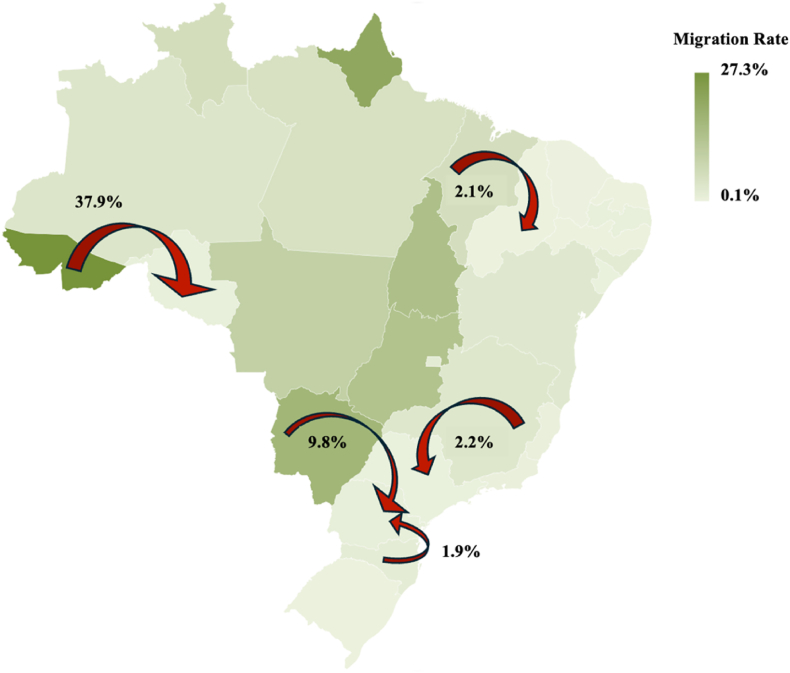
Migration rate for breast cancer treatment in Brazil (2017–2022).

Legend: This map illustrates the migration rate of breast cancer patients across Brazilian states for oncological treatment between 2017 and 2022. The shading intensity represents the migration rate (%) from each state, with darker tones indicating higher proportions of patients who sought treatment outside their home state. The map highlights significant migration flows towards the Southeast and South regions of Brazil.

Analysis of inter-state migration patterns for breast cancer treatment revealed a national average migration rate of 2.1 % (Table 2). The Northern region had the highest migration rate (3.9 %), with patients frequently traveling to the Southeast—particularly São Paulo—for treatment. Three Northern states exhibited strikingly high rates: Acre (43.1 %), Amapá (39.9 %), and Roraima (13.9 %), reflecting severe local limitations in treatment access. Notably, 37.9 % of Acre's patients migrated to neighboring Rondônia. The Midwest region showed the second highest regional migration rate (9.3 %), with Mato Grosso do Sul standing out at 18.3 %; most patients traveled to Paraná (9.8 %). In contrast, the Southeast region had a low overall migration rate (0.4 %), although Minas Gerais showed a slightly higher rate (2.7 %), primarily to São Paulo (2.2 %). The South reported the lowest regional migration rate (0.3 %), though Santa Catarina reached 2.3 %, with most patients heading to Paraná (1.9 %). The Northeast maintained a relatively low migration rate (0.3 %), with Maranhão presenting the highest intra-regional migration (2.8 %), mostly to Piauí (2.1 %). In Fig. 1 we find the maim migration rate for each region of Brazil.

**Table 2:** Migration rate by State and Region for Breast Cancer Treatment in Brazil.

Federative Unit	Migration Rate (%)	Most Common Migration Destination		Average Distance (km)
		Federative Unit	(%)	
**Brazil**	2.1	–	–	–

**Northern Region**	3.9	Southeast	3,2	–
Rondônia	5.5	São Paulo	4.9	2764
Acre	43.1	Rondônia	37.9	880
Amazonas	3.4	Rondônia	2,4	760
Roraima	13.9	Amazonas	7.9	663
Pará	4.7	São Paulo	0.7	2870
Amapá	39.9	Rondônia	6.1	3500
Tocantins	11.9	Sao Paulo	9.5	1814
**Midwest Region**	9.3	Southeast	6.6	–
Mato Grosso do Sul	18.3	Paraná	9.8	974
Mato Grosso	9.1	São Paulo	7.2	1532
Goiás	13.4	São Paulo	7.8	1088
Distrito Federal	2.2	Goiás	0.5	314
**Southeast Region**	0.4	Norte	0.2	–
Minas Gerais	2.7	São Paulo	2.2	593
Espírito Santo	0.9	Minas Gerais	0.5	523
Rio de Janeiro	0.8	Minas Gerais	0.3	439
São Paulo	0.7	Minas Gerais	0.1	593
**South Region**	0.3	Southeast	0.2	–
Paraná	1.3	Santa Catarina	0.7	305
Santa Catarina	2.3	Parana	1.9	305
Rio Grande do Sul	0.4	Santa Catarina	0.2	534
**Northeast Region**	0.3	Southeast	0.1	–
Maranhão	2.8	Piauí	2.1	453
Piauí	0.9	São Paulo	0.2	2656
Ceará	0.3	Sao Paulo	0.1	2654
Rio Grande do Norte	0.3	Paraíba	0.1	280
Paraíba	0.9	Pernambuco	0.4	118
Pernambuco	0.3	Bahia	0.1	846
Alagoas	2.2	Pernambuco	1.2	269
Sergipe	0.9	Pernambuco	0.3	511
Bahia	2.5	Pernambuco	1.3	846
				

Legend: The table categorizes breast cancer cases by Brazilian regions and states, indicating both the absolute number and the percentage of total cases within the specified period. To calculate distances considered as a reference point for the capitals of each federative unit.

Several notable patterns emerge from the migration data presented in Table 2. First, a clear North–South gradient is observed, with Northern states exhibiting significantly higher migration rates (regional average of 3.9 %) compared to the South (0.3 %). Second, smaller and more resource-limited states show disproportionately high migration rates—most notably Acre (43.1 %), Amapá (39.9 %), and Roraima (13.9 %)—underscoring stark inequalities in treatment accessibility. Third, migration trends reflect a consistent movement toward neighboring states with stronger healthcare infrastructure or toward major referral centers in the Southeast, particularly São Paulo. Geographical proximity plays a key role: patients from states with accessible neighboring services tend to travel shorter distances (e.g., Santa Catarina to Paraná, 305 km), whereas patients from more isolated states must travel far greater distances (e.g., Amapá to Rondônia, approximately 3500 km), further compounding barriers to timely treatment.

### 3.3 Migration rate by clinical staging for breast cancer treatment

Analysis of migration rates stratified by clinical stage revealed a progressive increase with advancing disease, ranging nationally from 0.4 % at stage 0–1.7 % at stage IV (p < 0.001) (Table 3). Regional disparities were pronounced, with the Northern region consistently showing the highest migration rates across all stages—most notably at stage IV (8.3 %, p < 0.001). Within this region, Acre presented exceptionally high migration at early stages, with 33.3 % at stage 0 and 49.0 % at stage I (both p < 0.001), while Amapá showed a dramatic stage IV migration rate of 91.7 % (p < 0.001), reflecting profound limitations in local treatment capacity.

**Table 3:** **–** Migration rate by Clinical Stage.

Federative Unit	Migration Rate by Clinical Stage (%)					p-value
	0	I	II	III	IV	
**Brazil**	0.4	0.9	1.4	1.1	1.7	<0.001

**Northern Region**	2.6	3.9	2.3	2.4	8.3	<0.001
Rondônia	0.0	0.9	0.0	0.8	1.3	0.544
Acre	33.3	49.0	35.3	18.1	27.5	<0.001
Amazonas	0.0	8.5	3.5	3.1	3.1	0.125
Roraima	0.0	6.9	8.9	1.3	9.3	0.295
Pará	40.0	7.1	3.6	2.7	20.5	<0.001
Amapá	2.0	22.5	12.9	63.2	91.7	<0.001
Tocantins	9.1	17.9	11.8	13.6	20.0	0,398
**Midwest Region**	3.4	11.4	10.7	7.4	11.9	<0.001
Mato Grosso do Sul	10.7	13.4	17.0	16.2	27.0	<0.001
Mato Grosso	6.7	9.4	6.8	7.6	20.6	<0.001
Goiás	3.9	18.2	16.7	14.1	11.7	<0.001
Distrito Federal	0.0	1.8	1.8	1.5	2.5	0.808
**Southeast Region**	0.2	0.4	0.3	0.3	0.3	0.096
Minas Gerais	0.9	4.1	2.5	2.7	5.6	<0.001
Espírito Santo	0.9	0.3	0.4	0.9	0.7	0.432
Rio de Janeiro	0.0	0.8	1.0	0.4	0.7	0.013
São Paulo	0.4	0.7	0.5	0.5	0.5	0.237
**South Region**	0.0	0.1	0.3	0.3	0.2	0.016
Paraná	0.2	0.9	1.0	1.3	0.8	0.087
Santa Catarina	1.4	1.1	2.2	1.5	1.0	0.023
Rio Grande do Sul	0.0	0.2	0.1	0.5	0.2	0.005
**Northeast Region**	0.0	0.3	0.2	0.2	0.7	<0.001
Maranhão	8.3	3.9	8.2	4.7	4.2	0.002
Piauí	0.0	0.0	0.2	0.2	0.0	0.847
Ceará	0.0	0.2	0.0	0.1	0.5	0.008
Rio Grande do Norte	0.8	0.0	0.1	0.2	0.5	0.272
Paraíba	0.0	0.8	0.9	0.6	0.7	0.907
Pernambuco	0.0	0.4	0.1	0.2	0.1	0.594
Alagoas	0.0	1.5	2.2	1.6	4.4	0.111
Sergipe	0.0	0.6	0.0	0.4	2.1	0.041
Bahia	2.7	1.9	3.1	2.7	4.2	0.013

Legend: This table shows the migration rates of breast cancer patients in Brazil, categorized by clinical staging and region from 2017 to 2022. The migration rate is presented as a percentage (%) and the statistical significance, indicated by the p-value, is derived from Chi-Square tests.

The Midwest region also demonstrated substantial migration at stages I (11.4 %), II (10.7 %), and IV (11.9 %) (all p < 0.001), with Mato Grosso do Sul (27.0 %) and Goiás (11.7 %) contributing significantly to the elevated stage IV migration (both p < 0.001). In contrast, the Southeast region showed consistently low migration rates across all stages (0.2 %–0.4 %), with the exception of Minas Gerais, which recorded a stage IV migration rate of 5.6 % (p < 0.001). The South region maintained overall low migration, though stage IV migration was modestly but significantly elevated (0.2 %, p = 0.016). Similarly, the Northeast region exhibited low migration rates overall, with a notable increase at stage IV (0.7 %, p < 0.001); Maranhão and Bahia had the highest regional rates at stages 0 and IV (p = 0.002 and p = 0.013, respectively).

### 3.4 Migration rate by treatment modality

Examination of migration patterns by treatment modality revealed substantial variation, with national migration rates of 7.8 % for surgery, 3.4 % for chemotherapy, and 17.5 % for radiotherapy (all p < 0.001) (Table 4). Within the Northern region, migration rates were 1.2 % for surgery, 3.2 % for chemotherapy, and 5.1 % for radiotherapy (p < 0.001). Notably, Roraima and Amapá both exhibited 100 % migration rates for radiotherapy (p < 0.001), indicating complete absence of local radiotherapy infrastructure. Acre demonstrated particularly high migration rates across all modalities: 2.5 % for surgery, 25.2 % for chemotherapy, and 63.2 % for radiotherapy (p < 0.001). The Midwest region showed elevated migration rates of 7.4 % for surgery, 9.5 % for chemotherapy, and 8.3 % for radiotherapy (p = 0.002), with Mato Grosso do Sul recording the highest regional rates across all modalities (22.3 %, 17.3 %, and 16.4 %, respectively; p = 0.013). The Southeast region maintained consistently low migration rates (all ≤0.3 %, p = 0.236), as did the South region (≤0.4 %, p = 0.058), though Santa Catarina exhibited higher rates for surgery (5.7 %) and chemotherapy (1.5 %) (p < 0.001). The Northeast region showed minimal migration (0.3–0.4 %, p = 0.393), with only Maranhão demonstrating notably higher radiotherapy migration (10.2 %, p = 0.076).

**Table 4:** Migration rate by treatment modality by state and region.

Federative Unit	Migration Rate by Treatment Modality (%)			p-value
	Surgery	Chemotherapy	Radiotherapy	
**Brazil**	7.8	3.4	17.5	<0.001

**Northern Region**	1.2	3.2	5.1	<0.001
Rondônia	1.5	0.6	1.0	0.480
Acre	2.5	25.2	63.2	<0.001
Amazonas	1.3	3.8	0.0	<0.001
Roraima	16.3	1.5	100	<0.001
Pará	2.4	4.9	3.1	0.003
Amapá	5.2	16.9	100	<0.001
Tocantins	4.2	14.4	12.2	<0.001
**Midwest Region**	7.4	9.5	8.3	0.002
Mato Grosso do Sul	22.3	17.3	16.4	0.013
Mato Grosso	3.4	9.9	6.8	<0.001
Goiás	10.8	13.9	10.3	0.005
Distrito Federal	1.8	1.7	1.3	0.961
**Southeast Region**	0.3	0.3	0.2	0.236
Minas Gerais	1.7	3.2	2.8	0.875
Espírito Santo	0.7	0.6	0.4	0.088
Rio de Janeiro	0.4	0.2	0.1	0.331
São Paulo	0.5	0.5	0.3	0.331
**South Region**	0.4	0.2	0.1	0.058
Paraná	1.3	1.1	0.2	0.032
Santa Catarina	5.7	1.5	1.8	<0.001
Rio Grande do Sul	0.1	0.3	0.1	0.248
**Northeast Region**	0.3	0.3	0.4	0.393
Maranhão	5.4	5.3	10.2	0.076
Piauí	0.5	0.2	0.0	0.586
Ceará	0.5	0.1	0.6	<0.001
Rio Grande do Norte	0.3	0.2	0.0	0.646
Paraíba	0.3	0.7	0.6	0.293
Pernambuco	0.5	0.2	0.3	0.058
Alagoas	2.7	2.0	1.1	0.691
Sergipe	1.0	0.5	0.0	0.463
Bahia	1.6	3.1	0.7	<0.001

Legend: This table details the migration rates of breast cancer patients by treatment modality (surgery, chemotherapy, radiotherapy) across different regions and states of Brazil. The migration rates are given as percentages, and the p-values, derived from Z-test two proportions, indicate the statistical significance of the differences observed.

The analysis by treatment modality reveals critical gaps in radiotherapy access, with a national migration rate of 17.5 % for this modality compared to 7.8 % for surgery and 3.4 % for chemotherapy. The most striking disparities are observed in Roraima and Amapá, where 100 % of patients requiring radiotherapy must migrate, indicating a complete absence of this treatment modality in these states. The Midwest region shows a more balanced migration pattern across treatment modalities (7.4 % for surgery, 9.5 % for chemotherapy, and 8.3 % for radiotherapy), suggesting more generalized healthcare infrastructure limitations rather than modality-specific gaps. In contrast, the South and Southeast regions demonstrate low migration rates across all treatment modalities (all below 1 %), highlighting their more comprehensive cancer care infrastructure.

### 3.5 Achievement of GBCI early detection threshold across Brazilian states

In alignment with the WHO's Global Breast Cancer Initiative (GBCI) Key Performance Indicator for early detection (Pillar 1), which establishes that at least 60 % of invasive breast cancer cases should be diagnosed at Stage I or II, our analysis revealed pronounced regional disparities in early diagnosis rates (Table 5, Supplementary Material). The national average of 56.2 % for Stage I and II diagnoses fell below the GBCI benchmark. Regional variation was substantial, with the South and Southeast regions generally performing better than other areas. São Paulo (67.8 %), Rio Grande do Sul (65.3 %), and Santa Catarina (63.9 %) all exceeded the 60 % threshold, while Minas Gerais (59.8 %) and Rio de Janeiro (58.7 %) approached but did not reach this benchmark. In stark contrast, most Northern states performed substantially below threshold, with Amapá (42.1 %) and Roraima (44.8 %) demonstrating the lowest proportions of early-stage diagnoses nationally. The Midwest region showed mixed performance, with only the Federal District (62.1 %) exceeding the threshold, while other states in the region ranged from 53.8 % to 57.3 %. Among Northeastern states, only Ceará (61.2 %) and Pernambuco (60.7 %) achieved the benchmark, with several states approaching but not reaching the threshold, including Bahia (56.8 %), Maranhão (55.2 %), and Rio Grande do Norte (57.3 %). These findings highlight significant geographic disparities in early detection capabilities across Brazil and identify regions requiring targeted intervention to improve early diagnosis rates.

**Table 5:** Distribution of breast cancer cases by clinical stage and achievement of GBCI 60 % threshold across Brazilian states (2017–2022).

Federative Unit	Stage 0-II (%)	Stage III-IV (%)	Meets GBCI Threshold
**Northern Region**	49.7	50.3	No
**Rondônia**	53.2	46.8	No
**Acre**	47.5	52.5	No
**Amazonas**	49.4	50.6	No
**Roraima**	44.8	55.2	No
**Pará**	51.2	48.8	No
**Amapá**	42.1	57.9	No
**Tocantins**	54.6	45.4	No
**Midwest Region**	56.3	43.7	No
**Mato Grosso do Sul**	54.5	45.5	No
**Mato Grosso**	53.8	46.2	No
**Goiás**	57.3	42.7	No
**Distrito Federal**	62.1	37.9	Yes
**Southeast Region**	61.8	38.2	Yes
**Minas Gerais**	59.8	40.2	No
**Espírito Santo**	57.3	42.7	No
**Rio de Janeiro**	58.7	41.3	No
**São Paulo**	67.8	32.2	Yes
**South Region**	63.7	36.3	Yes
**Paraná**	61.2	38.8	Yes
**Santa Catarina**	63.9	36.1	Yes
**Rio Grande do Sul**	65.3	34.7	Yes
**Northeast Region**	55.1	44.9	No
**Maranhão**	55.2	44.8	No
**Piauí**	53.5	46.5	No
**Ceará**	61.2	38.8	Yes
**Rio Grande do Norte**	57.3	42.7	No
**Paraíba**	55.9	44.1	No
**Pernambuco**	60.7	39.3	Yes
**Alagoas**	48.9	51.1	No
**Sergipe**	52.3	47.7	No
**Bahia**	56.8	43.2	No
**Brazil (National Average)**	56.2	43.8	No

## 4 Discussion

With its vast territory spanning approximately 8.5 million square kilometers, Brazil faces significant challenges in providing equitable access to breast cancer treatment. The country's immense geographical size, coupled with social inequality, exacerbates disparities in healthcare access, including early diagnosis, treatment, and follow-up services [14,15]. Regional variations and unequal health infrastructure significantly impact the availability and quality of services provided to patients across the nation.

Many patients are compelled to migrate to other regions to find appropriate professionals and treatment centers when local resources are lacking. Under the Brazilian Unified Health System (SUS), principles of regionalization and hierarchy guarantee cancer patients the right to receive treatment outside their municipality or state if necessary [16]. However, underreporting may occur as patients often provide alternative addresses to avoid being denied care [16]. Additionally, the migration to specialized centers introduces various challenges, such as health system saturation, long waiting lines, treatment delays, and the direct and indirect costs associated with migration.

A critical challenge is the uneven distribution of specialized cancer centers in Brazil. While large cities in the South and Southeast regions host most comprehensive hospitals and clinics with advanced infrastructure for breast cancer diagnosis and treatment, rural and remote areas suffer from significant resource limitations. This disparity forces many patients to travel long distances for adequate care, resulting in additional expenses and treatment delays [17].

Comparatively, the United States, another country of continental dimensions, boasts an advanced and comprehensive healthcare system with many specialized hospitals and efficient, organized health services, as seen in high-income countries compared to developing nations [18]. Similarly, China, another vast country, faces challenges similar to Brazil regarding access to breast cancer treatment. Residential status, as shown by Huo et al., 2015 [18], was a factor associated with treatment delays, where residents of large cities like Beijing and Shanghai, which house referral hospitals for cancer diagnosis and treatment, had lower chances of treatment delays, while patients in rural and remote areas had limited availability and quality of medical services [19,20].

Despite differences in healthcare systems and medical infrastructure, all these countries face challenges related to access to breast cancer treatment due to their continental dimensions. These challenges stem from geographical distances, uneven distribution of medical resources across regions, and financial barriers to accessing necessary care [17].

The challenges of breast cancer treatment access in Brazil mirror those observed in other middle-income countries with large territories. In Mexico, a study by Unger-Saldaña et al. [21] found that geographical barriers were responsible for significant treatment delays, with patients from rural areas experiencing 2.4 times longer waits than urban dwellers. Similarly, in South Africa, Dickens et al. [22] reported that patients traveling from distant areas to urban treatment centers experienced delays averaging 8.5 weeks longer than local patients.

In India, a country with comparable geographical challenges, Neal et al. [23] documented that patients traveling more than 200 km for radiotherapy faced a 38 % greater risk of treatment discontinuation compared to those receiving care within their region. The radiotherapy access issues we observed in Brazil's Northern states (with 100 % migration rates in Roraima and Amapá) parallel findings from a national assessment in Turkey, where Sezer et al. reported that five of the country's 81 provinces had no radiotherapy facilities, resulting in migration rates exceeding 95 % for this treatment modality.

A systematic review by examining healthcare access in several middle-income countries found that breast cancer patients who migrated for treatment had a 1.5–2.3 times higher risk of experiencing treatment discontinuation compared to those treated locally, highlighting how migration rate can impact treatment completion [24,25].

The migration rate patterns documented in our study have profound implications for patient outcomes. Treatment delays resulting from migration can significantly impact survival rates, particularly for advanced-stage breast cancer. A meta-analysis by Neal et al. [23] found that each month of delay in treatment initiation was associated with a 1.8 % decrease in overall survival for stage III and IV breast cancer.

Beyond survival, migration-related delays affect quality of life and treatment adherence. Patients who travel long distances for treatment often experience financial hardship, accommodation challenges, family separation, and psychological distress. These factors can lead to treatment abandonment or non-adherence. Studies from Colombia and Malaysia have documented treatment abandonment rates of 18 % and 22 %, respectively, among cancer patients who migrated for care, compared to rates of 7 % and 9 % among local patients [24].

The observed high migration rates for radiotherapy in our study are particularly concerning given the protracted nature of this treatment (typically 5–6 weeks). Incomplete radiotherapy regimens resulting from the hardships of long-term relocation may compromise locoregional control and ultimately affect survival. In Brazil, a study found that breast cancer patients who received incomplete radiotherapy courses due to logistical challenges had a 24 % higher risk of locoregional recurrence over a five-year follow-up period [26].

In Brazil, specifically regarding radiotherapy, the disparity in access and availability of this infrastructure is exacerbated, as there are still regions where access to radiotherapy treatment is limited or even nonexistent [21]. States in the North Region, such as Roraima and Amapá, lack centers with access to radiotherapy. Consequently, 100 % of patients from these states must travel to others to seek appropriate treatment, as shown in this study. Other regions in the country may also face difficulties accessing radiotherapy, especially rural areas and those far from major urban centers, as equipment availability is directly linked to investments and resources allocated to health in each state.

Regarding chemotherapy, in 2015, SUS performed 2,644,897 treatments nationwide, including neoadjuvant, adjuvant, and palliative chemotherapy. São Paulo accounted for 708,335 procedures, representing 26.8 % of the total [27]. In contrast, Amapá had the lowest number of procedures, with only 1610 (0.1 % of the total). These numbers are directly related to the population of each state, but also to the overload of demands from other locations. The Federal District has the highest percentage of out-of-state patients (17 % from Goiás and 2 % from Minas Gerais), while Roraima has the lowest rate of out-of-state patient care [10,27].

A deleterious effect of the high migration rate of patients to major medical centers for various breast cancer treatments is the saturation of these institutions [28]. Particularly in the South and Southeast regions, which concentrate reference hospitals and clinics with advanced infrastructure and specialists. These locations are sought after by patients from all over the country, seeking specialized care and access to more advanced treatments.

However, the size of the hospital and the high demand in these institutions can overwhelm the capacity for care and the available infrastructure [29,30]. This can result in long waiting lines, delays in scheduling consultations, exams, and diagnostic and therapeutic procedures. Overcrowding can lead to delays in the start of treatment, which can be detrimental to the prognosis of breast cancer patients. Medeiros et al., 2023 [17] show the contradiction of the country, where patients diagnosed and treated in high-complexity centers took longer to start treatment.

Moreover, not all patients have the financial means or capacity to travel to other regions of the country for care, creating disparities in access to health, adequate treatment, and its timely initiation [17]. In middle- and low-income countries, the lack of access to health services can lead to longer intervals and, according to some authors, the long interval from patients to specialists and the long total interval (diagnosis and treatment interval) most impact the disease prognosis [18]. The distance to specialized centers is identified as one of the main factors contributing to long intervals [18,30]. Patients living in rural or remote areas need to travel long distances for treatment, delaying its initiation. According to Oliveira et al., 2011 [24], between 2005 and 2006, a high percentage of breast cancer patients in Brazil, treated under SUS, traveled more than 150 km from their homes to the treatment location.

Five-year survival rates for breast cancer are significantly lower in low- and middle-income countries such as Brazil, India, Algeria, and Gambia, compared to countries like the United States, Sweden, Japan, and Australia [31,32]. This is due to the lack of early detection programs and adequate diagnostic and treatment facilities, as well as treatment delays.

In light of this, aiming to optimize the onset of oncological treatment in the country, the Brazilian Ministry of Health established Law 12.732/12 in 2012, which aims to ensure access to treatment within 60 days from the histopathological report [17]. However, the effective implementation of the law only occurred in 2013 with Ordinance 876/13.

Comparatively, in high-income countries, the healthcare system is generally organized and efficient, resulting in shorter intervals [30,31], such as in the USA, which has treatment initiation indices between 22 and 27 days after diagnosis, and Canada, with an average of 34 days until treatment begins. However, disparities in access also exist in these countries, mainly due to socioeconomic differences and the health insurance system, unlike Brazil where there is the possibility of universal access. In major centers in Brazil, the average time index was over 70 days in the South and Southeast [19].

On the other hand, although there is concern about time intervals in the healthcare system, studies indicate that longer intervals do not have a significant adverse impact on the survival of breast cancer patients [[33], [34], [35]].

Furthermore, the direct and indirect financial difficulties associated with migration rate and treatment can lead to inadequate adherence. Between 21 % and 44 % of cancer patients and survivors face financial difficulties during the disease [35]. Few studies are available on cancer survivors with a migration rate history, but a German study showed that these survivors are more likely to face financial difficulties during the disease [34]. Additionally, the same study shows that compared to non-migrants, breast cancer survivor patients with a migration rate history were almost four times more likely to experience financial difficulties in the final stages of treatment and follow-up [34].

Our analysis of early-stage diagnosis rates in relation to the GBCI benchmark reveals a concerning pattern of regional inequality in Brazil's breast cancer detection capabilities. The achievement of the 60 % threshold for Stage I and II diagnoses in the more affluent South and Southeast regions, contrasted with poorer performance in Northern and many Northeastern states, mirrors broader socioeconomic disparities in the country. These findings align with Duggan et al. [5] observation that health system characteristics strongly influence stage at diagnosis and subsequent mortality outcomes. The states falling below the GBCI benchmark likely face multiple challenges, including insufficient screening infrastructure, limited access to diagnostic services, lower awareness among the population, and shortages of trained healthcare professionals specializing in breast cancer detection. This situation demands targeted intervention from Brazil's Ministry of Health, with resources and programs specifically designed to strengthen early detection capabilities in underperforming regions. Without such focused initiatives, these regional disparities will continue to drive inequitable breast cancer outcomes across the country.

In Brazil, as an attempt to assist with this issue, oncology patients may benefit from the Out-of-Home Treatment (TFD) program [[36], [37], [38]] if they have exhausted treatment options in their region, are receiving treatment through SUS, and have a distance of over 50 km between their residence and the treatment center. TFD provides financial assistance for food, air transport, river transport, and land transportation expenses for both the patient and the companion.

The cost of breast cancer treatment varies significantly depending on the stage of the disease, starting at just over R$ 11,000 (US$ 3300) in the early stages and rising to approximately R$ 55,000 (US$ 16,500) by stage III. The average total treatment cost for neoadjuvant or adjuvant therapy is R$ 14,497.70 (US$ 4349.31), while palliative treatment averages R$ 9108.60 (US$ 2732.58). Tamoxifen represents the largest component of treatment costs, accounting for over 80 % of the total, with an annual average cost of R$ 1947.60 (US$ 584.28) per patient. In some cases, treatment costs can reach as high as R$ 93,241.01 (US$ 27,972.30) [36].

These figures highlight that the average cost of treatment is high compared to the average Brazilian salary of R$ 2110.00 (US$ 633.00) (IBGE-2017) [38]. When comparing different surgical approaches, the costs are generally comparable. The reduction in hospitalization time and faster recovery associated with breast-conserving surgery can offset the costs of radiotherapy for these patients [39,40].

Access to breast cancer treatment in Brazil is challenged by the country's vast size, regional inequalities in healthcare infrastructure, and limited availability of medical resources. These geographical disparities, marked by the uneven distribution of specialized medical centers, force many patients to travel long distances for adequate care, leading to treatment delays and adversely affecting disease outcomes. Addressing these challenges requires a concerted effort by the Brazilian healthcare system to implement comprehensive strategies aimed at reducing regional inequalities in access to breast cancer treatment. Key measures include decentralizing healthcare services, launching screening and awareness programs in remote areas, improving medical infrastructure in underserved regions, and ensuring that high-quality care is accessible to all, regardless of geographic location or socioeconomic status.

The findings of this study are particularly relevant when viewed through the lens of the WHO's Global Breast Cancer Initiative (GBCI) [5,6,9]. Brazil's Law 12.732/12, which mandates treatment initiation within 60 days of diagnosis, aligns with the GBCI's Pillar 2, emphasizing the importance of timely diagnosis and care [7]. However, our results highlight significant challenges in fulfilling Pillar 3 (treatment completion), with high migration rates for radiotherapy in several states indicating substantial barriers to comprehensive care. The observed regional disparities in accessing treatment reflect the challenges of implementing global health frameworks in countries with vast geographical and socioeconomic differences [8].

From a policy standpoint, our findings highlight the urgent need for Brazil to enhance the implementation of the GBCI framework [7,9]. In response to the global, with particular emphasis on expanding radiotherapy infrastructure in underserved areas to ensure treatment completion (Pillar 3). Furthermore, the observed differences in patient migration by clinical stage indicate that early detection efforts (Pillar 1) should be strategically intensified in regions with a higher prevalence of advanced-stage diagnoses—especially in the Northern and Northeastern states—in alignment with GBCI recommendations [9].

An important limitation is that our study only captures data on patients who successfully accessed the healthcare system and had their information recorded in the DATASUS database. This creates a potential selection bias, as patients with the most limited access to healthcare—often those with advanced disease in remote areas—may not be represented in the dataset [8]. This underrepresentation could skew stage distribution statistics, potentially underestimating the proportion of advanced-stage diagnoses. The true burden of late-stage breast cancer in underserved regions may therefore be greater than what our analysis suggests, further emphasizing the need for improved healthcare access and early detection initiatives aligned with GBCI Pillar 1 [7,9].

### 4.1 Feasible policy recommendations based on international Best practices

Our findings suggest several concrete policy measures that could address the geographical disparities in breast cancer treatment access in Brazil:

1. **Expansion of Radiotherapy Infrastructure**: Our data showing 100 % migration rates for radiotherapy in some states highlights the critical need for infrastructure development. Based on successful models from Thailand and Turkey, we recommend a phased expansion of radiotherapy facilities, prioritizing states with the highest migration rates. The International Atomic Energy Agency (IAEA) recommends at least one radiotherapy unit per 250,000 population [41]; currently, the North and Northeast regions of Brazil fall significantly below this standard.
2. **Telemedicine and Remote Case Management**: Drawing on Colombia's successful telemedicine initiatives [42], Brazil could implement a national telemedicine network connecting oncologists in reference centers with healthcare professionals in underserved areas. This would facilitate remote treatment planning, follow-up consultations, and reduce unnecessary travel while ensuring quality care.
3. **Patient Navigation Programs**: Structured patient navigation programs, similar to those implemented in Mexico [43], could help patients from remote areas navigate the complex healthcare system more effectively. Such programs have reduced treatment abandonment rates by 40 % among patients traveling for cancer care.
4. **Decentralization of Chemotherapy Services**: Following Mexico's successful model of chemotherapy decentralization [43], Brazil could establish satellite chemotherapy units in high-migration rate regions, linked to comprehensive cancer centers for quality assurance. These units require less infrastructure than full oncology centers but can significantly reduce migration rate for chemotherapy.
5. **Enhanced Patient Support Systems**: Expanding the existing Out-of-Home Treatment (TFD) program to include comprehensive support such as designated patient housing near treatment facilities and transportation assistance programs would address important barriers to care for migrating patients [44].
6. **Mobile Screening and Early Detection Units**: To address the lower rates of early-stage diagnoses in Northern and Northeastern regions, mobile screening units (successfully implemented in Brazil's more affluent regions) could improve access to mammography and clinical breast exams in underserved areas [26].

The successful implementation of these recommendations requires coordinated action across federal, state, and municipal levels, as well as adequate funding allocation. The economic benefits of reduced migration-related costs and improved treatment outcomes should be emphasized to policymakers as justification for these investments.

## 5 Conclusions

The migration rate of breast cancer patients in Brazil for treatment highlights significant regional disparities in healthcare access. These inequities emphasize the urgent need for targeted health policies aimed at ensuring equitable access to cancer treatment services across all regions. The observed trends point to the critical importance of strengthening healthcare infrastructure in under-resourced areas and expanding the availability of specialized treatment centers to minimize the need for patient migration. Addressing these issues is essential for improving treatment outcomes and ensuring timely, effective care for breast cancer patients nationwide.

## Authors' contributions

(I) Conception and design: Marcelo Antonini, Andre Mattar(II) Administrative support: Marcelo Antonini, Andre MattarORCID, Odair Ferraro, Luis Henrique Gebrim, Marina Diógenes Teixeira, Andressa Gonçalves Amorim, Juliana Francisco, (III) Provision of study materials or patients: Marcelo Antonini, Gabriela Moreira Santos, Felipe Zerwes, Fabricio Palermo Brenelli, Marina Fleury de Figueiredo (IV) Collection and assembly of data: Marcelo Antonini, Gabriela Moreira Santos (V) Data analysis and interpretation: Marcelo Antonini, Andre Mattar, Francisco Pimentel Cavalcante, Eduardo Camargo Millen, Antonio Luis Frasson, Marcellus do Nascimento Moreira Ramos (VI) Manuscript writing: All authors. (VII) Final approval of manuscript: All authors.

## Ethical approval

As this is an ecological study, it is exempt from requiring.

## Funding

No funding

## Declaration of competing interest

The authors declare that they have no known competing financial interests or personal relationships that could have appeared to influence the work reported in this paper.
