# Supplementary material for: Barriers to breast cancer treatment in Brazil: A study on migration and regional disparities
Source: Public Health Pract (Oxf). 2025 May 13;9:100614. doi: 10.1016/j.puhip.2025.100614 (PMC12148374; doi:10.1016/j.puhip.2025.100614)
Supplement: Multimedia component 1 [file mmc1.docx]

**Supplementaty Material**

Our analysis revealed significant regional disparities in early diagnosis rates. The South and Southeast regions generally performed better, with São Paulo (67.8%), Rio Grande do Sul (65.3%), and Santa Catarina (63.9%) exceeding the 60% threshold. In contrast, most Northern states fell below this benchmark, with Amapá (42.1%) and Roraima (44.8%) showing the lowest proportions of early-stage diagnoses. Among Northeastern states, only Ceará (61.2%) and Pernambuco (60.7%) achieved the threshold. These findings highlight areas where focused early detection initiatives are most urgently needed to improve breast cancer outcomes and align with GBCI Pillar 1 objectives [3,4]. This results we can find in the supplementary material (Table S1)

# Table S1: Distribution of Breast Cancer Cases by Clinical Stage and Achievement of GBCI 60% Threshold Across Brazilian States (2017-2022)

| **Federative Unit** | **Stage 0-II (%)** | **Stage III-IV (%)** | **Meets GBCI Threshold** |
| --- | --- | --- | --- |
| **Northern Region** | 49.7 | 50.3 | No |
| **Rondônia** | 53.2 | 46.8 | No |
| **Acre** | 47.5 | 52.5 | No |
| **Amazonas** | 49.4 | 50.6 | No |
| **Roraima** | 44.8 | 55.2 | No |
| **Pará** | 51.2 | 48.8 | No |
| **Amapá** | 42.1 | 57.9 | No |
| **Tocantins** | 54.6 | 45.4 | No |
| **Midwest Region** | 56.3 | 43.7 | No |
| **Mato Grosso do Sul** | 54.5 | 45.5 | No |
| **Mato Grosso** | 53.8 | 46.2 | No |
| **Goiás** | 57.3 | 42.7 | No |
| **Distrito Federal** | 62.1 | 37.9 | Yes |
| **Southeast Region** | 61.8 | 38.2 | Yes |
| **Minas Gerais** | 59.8 | 40.2 | No |
| **Espírito Santo** | 57.3 | 42.7 | No |
| **Rio de Janeiro** | 58.7 | 41.3 | No |
| **São Paulo** | 67.8 | 32.2 | Yes |
| **South Region** | 63.7 | 36.3 | Yes |
| **Paraná** | 61.2 | 38.8 | Yes |
| **Santa Catarina** | 63.9 | 36.1 | Yes |
| **Rio Grande do Sul** | 65.3 | 34.7 | Yes |
| **Northeast Region** | 55.1 | 44.9 | No |
| **Maranhão** | 55.2 | 44.8 | No |
| **Piauí** | 53.5 | 46.5 | No |
| **Ceará** | 61.2 | 38.8 | Yes |
| **Rio Grande do Norte** | 57.3 | 42.7 | No |
| **Paraíba** | 55.9 | 44.1 | No |
| **Pernambuco** | 60.7 | 39.3 | Yes |
| **Alagoas** | 48.9 | 51.1 | No |
| **Sergipe** | 52.3 | 47.7 | No |
| **Bahia** | 56.8 | 43.2 | No |
| **Brazil (National Average)** | 56.2 | 43.8 | No |
